# Prevalence and risk factors of cognitive frailty in patients with cardiovascular disease: A hospital-based cross-sectional study

**DOI:** 10.1097/MD.0000000000040761

**Published:** 2024-12-06

**Authors:** Jinhua Guo, Yi Zhang, Yi Yang, lixia Lin, Tiemei Shen

**Affiliations:** a Department of Cardiovascular Disease Institute, Guangdong Provincial People’s Hospital (Guangdong Academy of Medical Sciences) Southern Medical University, Guangzhou, China.

**Keywords:** cardiovascular disease, cognitive frailty, elderly, influencing factors

## Abstract

The prevalence of the cognitive frailty is increasing in China. Screening for this condition is crucial for its early detection, prevention, and treatment. This study was designed to explore the incidence of cognitive frailty among hospitalized elderly patients suffering from cardiovascular disease. It also aimed to analyze the factors influencing its occurrence, thereby providing substantial evidence for the development of early prevention and intervention strategies.

From March 2022 to October 2023, under cardiovascular care program, the cardiovascular patients (n = 1190) were subjected to standardized questionnaires to collect demographical characteristics. Also, nutritional and psychosocial assessments tests were performed for the enrolled patients. Multivariate logistic regression analysis was used to evaluate factors associated with cognitive frailty.

A total of 1190 (755 males and 435 females) were included. The mean age was 73.36 ± 7.37 years. The prevalence of cognitive frailty in the study population was 33.9% (404/1190). The prevalence of cognitive frailty was 40.7% in men, 22.3% in women. In terms of specific cardiovascular diseases, the prevalence of cognitive frailty was 28.5% in coronary heart disease, 20.5% in arrhythmia, 36.8% in valvular disease, 53% in heart failure, and 13.7% in hypertension. The multivariable analysis showed that age (OR = 1.13, 95% CI: 1.10–1.15, *P* < .001), anxiety (OR = 1.01, 95% CI: 1.03–1.11, *P* = .001), female sex (OR = 1.83, 95% CI: 1.10–1.16, *P* < .001), education level (college and above, OR = 0.27, OR = 0.12–0.64, *P* = .003), polypharmacy (OR = 2.29, 95% CI: 1.62–3.23, *P* < .001), comorbidity (OR = 1.93 95% CI: 1.37–2.71, *P* < .010), region (rural, OR = 1.77, 95% CI: 1.36–2.30, *P* < .001), sarcopenia (OR = 1.60, 95% CI: 1.16–2.19, *P* = .004), and nutritional status (risk of malnutrition, OR = 1.66, 95% CI: 1.17–2.35, *P* = .004; malnutrition exists, OR = 3.24, 95% CI: 1.85–5.83, *P* < .001) were independently associated with cognitive frailty.

The prevalence of cognitive frailty was 33.9% in hospitalized elderly cardiovascular patients in Guangzhou. heart failure, hypertension, age, anxiety, female sex, education level, polypharmacy, comorbidity, region, sarcopenia, and nutritional status were independent risk factors for cognitive frailty.

## 1. Introduction

As the population ages, cardiovascular disease has risen to become the primary cause of death globally.^[1]^ Individuals with cardiovascular disease frequently exhibit multiple concurrent conditions, generally poor health, and are prone to various geriatric syndromes such as frailty and cognitive impairment.^[2]^ Frailty is a nonspecific condition characterized by a decline in the physiological reserves of the elderly, leading to increased vulnerability and diminished resistance to stress.^[3]^ It includes physical frailty, cognitive frailty, and socio-psychological frailty.^[4]^ Cognitive frailty is defined as the simultaneous presence of frailty and mild cognitive impairment.^[5]^

Epidemiological research indicates that cognitive frailty can hasten the deterioration of cognitive and physical functions in elderly individuals with cardiovascular disease. This deterioration can result in a diminished capacity to carry out daily activities, a decrease in quality of life, and an elevated risk of dementia, falls, disability, and death.^[6,7]^ Cognitive frailty, a reversible condition, requires early detection and effective interventions to slow its progression and avert adverse outcomes.^[8,9]^

However, in our country, there is a significant lack of research concerning the factors that contribute to the onset of cognitive frailty in hospitalized elderly patients with cardiovascular disease. Therefore, the purpose of this study is to examine the current state of cognitive frailty and its influencing factors in this specific patient group. The goal is to identify high-risk groups for cognitive frailty at an early stage and intervene against the associated risk factors.

## 2. Materials and methods

### 2.1. Study design and subjects

This was a cross-sectional study. From March 2022 to October 2023, the convenience sampling method was utilized to select elderly patients who were hospitalized in the Cardiovascular Disease Research Institute of Guangdong Provincial People’s Hospital as the research participants.

The inclusion criteria were:

1. ≥60 years old;
2. Clearly diagnosed cardiovascular diseases including coronary heart disease, hypertension, arrhythmia, heart failure, and heart valve disease;
3. Voluntary participation in this study and signing of an informed consent form. The exclusion criteria were
  1. Diagnosed dementia and cognitive impairment caused by other clear causes (vascular dementia, epilepsy, etc);
  2. Mental illnesses such as schizophrenia or abuse of psychoactive drugs;
  3. Severe visual, auditory, and limb functional impairments;
  4. Patients with severe diseases and terminal illnesses.

The study was approved by the Ethics Committee of Guangdong Provincial People’s Hospital (KY-N-2021-003-01 and KY-Z-2022-333-01). Informed consent was obtained from all subjects. This work has been carried out in accordance with the Declaration of Helsinki (2000) of the World Medical Association.

### 2.2. Sample size calculation

According to the sample size calculation formula,^[10]^ it is recommended to have 5~10 cases for each independent variable. In this study, there were 22 independent variables, so the sample size was calculated to be 10 times the number of independent variables. Taking into account a 20% loss to follow-up rate, it was estimated that 275 cases would be needed, and ultimately, 1190 cases were included.

### 2.3. Research instruments

1. General information survey form: This self-compiled form includes details such as age, gender, body mass index, education level, living conditions, occupation, type of medical insurance, average monthly family income, types of medication, smoking and drinking habits, medication usage, comorbidity, trauma events, and history of falls.
2. Physical frailty assessment: The FRAIL scale,^[11]^ proposed by experts from the International Nutrition and Aging Association in 2008. It covers 5 aspects and has a total score of 5 points. A score of 1 to 2 points indicates pre-frailty, and a score of ≥3 points indicates frailty.^[11–13]^
3. Mini-mental state examination (MMSE): This was compiled by Folstein et al^[14]^ in 1975, and translated and localized by Zhang MY et al.^[15]^ The total score is the sum of the scores of each item, with a score range of 0 to 30 points. The score is closely related to the level of education, and the criteria for cognitive impairment are: illiterate group ≤ 17 points, primary school education group ≤ 20 points, junior high school and above education group ≤ 24 points.
4. Sleep assessment: The Pittsburgh sleep quality index (PSQI) was used for evaluation. PSQI compiled by Buysee et al^[16]^ in 1989, with a score range of 0 to 21 points. A PSQI score > 7 points indicates the presence of sleep problems. Research by Chinese scholar Liu XC et al^[17]^ shows that it has high reliability and validity, with a Cronbach α coefficient of 0.84.
5. Nutritional status: The mini nutritional assessment short-form (MNA-SF) was simplified by Rubenstein et al^[18]^ in 2001 from the 18-item MNA, Scores of 12 to 14 points indicate normal nutritional status, 8 to 11 points indicate risk of malnutrition, and ≤ 7 points indicate malnutrition.
6. Sarcopenia: The SARC-F questionnaire was used for quick screening of sarcopenia, including slow walking, weakness, getting up from a chair or bed, climbing stairs, falling, etc. A score of 0 to 3 points indicates a very low risk of sarcopenia, and ≥4 points indicates the presence of sarcopenia.
7. Generalized anxiety disorder (GAD-7) scale was used to measure the anxiety state of the research subjects. Scores of 0 to 4 points indicate no anxiety; 5 to 9 points indicate mild anxiety; 10 to 14 points indicate moderate anxiety; 15 to 21 points indicate severe anxiety. The Cronbach α coefficient of GAD-7 is 0.898.^[19]^
8. Patient health questionnaire (PHQ-9) was used to measure the depression state of the research subjects. Scores of 0 to 4 points indicate no depression; 5 to 9 points indicate depressive symptoms; 10 to 14 points indicate obvious depressive symptoms; 15 to 27 points indicate severe depression. The Cronbach α coefficient of PHQ-9 is 0.767.^[20]^
9. Social support rating scale designed by Chinese scholar Xiao Shuiyuan^[21]^ in 1986, allows for analysis based on the total score and each dimension score. The internal consistency Cronbach α coefficient of this scale is between 0.82 and 0.896.
10. Barthel index: this index evaluates daily living ability, assessing the elderly’s self-care ability in daily life from ten aspects. The total score is 100 points, with a lower score indicating weaker self-care ability and a higher need for assistance.^[22]^

### 2.4. Relevant indicators and definitions

Cognitive frailty, as defined by the International Gerontology and Geriatric Medicine Expert Group in 2013.^[5]^ encompasses physical frailty and cognitive impairment, excluding all types of dementia. In this study, cognitive frailty is identified by scoring ≥1 on the frailty screening scale and rating MMSE as cognitive impairment. Polypharmacy is defined as taking 5 or more types of medications, while comorbidity is defined as having 2 or more chronic diseases concurrently.

### 2.5. Statistical methods

Data were analyzed using SPSS 26 (IBM Corporation, Armonk, New York). Normal distribution tests were performed on all continuous variables using the Kolmogorov–Smirnov test. Continuous data with a normal distribution were presented as means ± standard deviations. Continuous data with a skewed distribution were presented as medians (interquartile range, IQR). Categorical data were presented as n (%). The chi-square test was used to analyze the prevalence of cognitive frailty among different groups. The risk factors were analyzed by univariable and multivariable logistic regression analyses. The indicators with *P* < .1 in the univariable analyses were selected for inclusion. Two-sided *P*-values < .05 were considered statistically significant.

## 3. Results

### 3.1. Characteristics of the subjects

A total of 1215 hospitalized cardiovascular disease patients, ≥ 60 years of age, were selected as the study subjects. The actual number of subjects who completed all investigations was 1190 (755 males and 435 females), for a response rate of 97.9%. Table 1 presents the characteristics of the subjects. The mean age was 73.36 ± 7.37 years.

**Table 1:** Characteristics of the subjects.

Variable	Description	Value (N = 1190)
Age	Mean ± SD	73.36 ± 7.37
	Median (IQR)	74 (67, 78)
Sex	Male	755 (63.5%)
	Female	435 (36.5%)
Education level	Illiteracy	453 (38.1%)
	Primary school and below	472 (39.6%)
	Junior or senior high school	201 (16.8%)
	College and above	64 (5.3%)
Marital status	Married	988 (83.0%)
	Widowed	180 (15.2%)
	Others	22 (1.8%)
Type of medical insurance	Public medical	4 (0.3%)
	Medical insurance	1116 (93.7%)
	Business insurance	28 (2.4%)
	Others	42 (3.6%)
Family income per month, CNY	<1000	12 (1.1%)
	1000 to 2000	244 (20.5%)
	2000 to 5000	454 (38.1%)
	>5000	480 (40.3%)
Polypharmacy	Yes	710 (59.7%)
	No	480 (40.3%)
Comorbidity	Yes	704 (59.2%)
	No	456 (40.8%)
BMI, kg/m^2^	Mean ± SD	21.92 ± 3.58
	Median (IQR)	22.04 (19.4, 24)
Pre-retirement occupation.	Institutions	242 (20.3%)
	Foreign companies	28 (2.3%)
	Freelance business	346 (29.1%)
	Farmers.	574 (48.3%)
Region	County or town	413 (34.8%)
	Rural	777 (65.2%)
Smoking	Always	136 (11.4%)
	Occasional	646 (54.3%)
	Never	408 (34.3%)
Drinking	Always	40 (3.3%)
	Occasional	886 (74.4%)
	Never	264 (22.1%)
Traumatic event	Yes	36 (3.1%)
	No	1154 (96.9%)
History of falls	Yes	62 (5.2%)
	No	1128 (94.8%)
Surgical history.	Yes	142 (11.9%)
	No	1048 (88.1%)
Nutritional status.	Normal nutrition	758 (63.7%)
	Risk of malnutrition	337 (28.4%)
	Malnutrition exists	95 (7.9%)
Sarcopenia	Yes	514 (43.2%)
	No	676 (56.8%)
PSQI	Mean ± SD	9.38 ± 4.10
	Median (IQR)	8 (6, 13)
ADL	Mean ± SD	73.76 ± 18.19
	Median (IQR)	75 (60, 90)
GAD-7	Mean ± SD	5.43 ± 3.33
	Median (IQR)	5 (3, 8)
PHQ-9	Mean ± SD	6.15 ± 3.47
	Median (IQR)	6 (4, 8)
SSRS	Mean ± SD	27.63 ± 5.21
	Median (IQR)	28 (25, 32)

Abbreviations: ADL = activities of daily living, BMI = body mass index, GAD-7 = generalized anxiety scale, IQR = interquartile range, PHQ-9 = depression screening scale, PSQI = Pittsburgh sleep quality index, SD = standard deviation, SSRS = social support rating scale.

### 3.2. Prevalence of cognitive frailty

Table 2 presents the prevalence of cognitive frailty. The total prevalence of cognitive frailty in the study population was 33.9% (404/1190). The prevalence of cognitive frailty was 40.7% in men, 22.3% in women. In terms of specific cardiovascular diseases, the prevalence of cognitive frailty was 28.5% in coronary heart disease, 20.5% in arrhythmia, 36.8% in valvular disease, 53% in heart failure, and 13.7% in hypertension.

**Table 2:** Prevalence of cognitive frailty.

	Prevalence
Total	404/1190 (33.9%)
Gender	
Male	307/755 (40.7%)
Female	97/435 (22.3%)
Cardiovascular disease	
Coronary heart disease	174/609 (28.5%)
Arrhythmia	22/107 (20.5%)
Valvular heart disease	53/144 (36.8%)
Heart failure	148/279 (53.0%)
Hypertension	7/51 (13.7%)

### 3.3. Univariable regression analyses of cognitive frailty

The univariable analyses (Table 3) showed that age (OR = 1.13, 95% CI: 1.11–1.16, *P* < .001), PSQI (OR = 1.06, 95% CI: 1.03–1.10, *P < *.001, ADL (OR = 0.97, 95% CI: 0.96–0.97, *P* < .001), GAD-7 (OR = 1.17, 95% CI: 1.13–1.22, *P* < .001), PHQ-9 (OR = 1.10, 95% CI: 1.061.14, *P* < .001), female sex (OR = 2.39, 95% CI: 1.83–3.12, *P* < .001), education level (primary school and below, OR = 0.71, 95% CI: 0.54–0.93, *P* < .001; junior or senior high school, OR = 0.48, 95% CI: 0.34–0.70, *P* < .001; college and above, OR = 0.23, OR = 0.11–0.48, *P* < .001),family income per month, CNY (1000~2000, OR = 0.35, 95% CI: 0.11–1.13, *P* = .08); >5000, OR = 0.35, 95% CI: 0.11–1.11, *P* = .075), polypharmacy (OR = 3.07, 95% CI: 2.34–4.01, *P* < .001), comorbidity (OR = 2.33, 95% CI: 1.80–3.01, *P* < .001), Rural (OR = 1.77, 95% CI: 1.36–2.30, *P* < .001), sarcopenia (OR = 2.53, 95% CI: 1.98–3.23, *P* < .001), nutritional status (risk of malnutrition, OR = 1.91, 95% CI: 1.46–2.50, *P* < .001; malnutrition exists, OR = 4.87, 95% CI: 3.12–7.64, *P* < .001) were associated with cognitive frailty. The results show that general demographic social information on age, gender, literacy, economic income, multiple medication use, comorbidities and psychiatric-psychological status, sleep and nutrition are associated with cognitive frailty.

**Table 3:** Univariable regression analyses of cognitive frailty.

Variable	OR	95% CI	*P*
Age	**1.133**	**(1.111, 1.156**)	**<.001** *
BMI	0.998	*(0.965, 1.032*)	0.917
PSQI	**1.064**	***(1.033, 1.095***)	**<.001** *
ADL	**0.966**	**(0.959, 0.973**)	**<.001** *
GAD-7	**1.169**	**(1.125, 1.215**)	**<.001** *
PHQ-9	**1.099**	**(1.059, 1.137**)	**<.001** *
SSRS	0.996	(0.973, 1.019)	0.716
Sex			
Male	Reference		
Female	**2.388**	**(1.826, 3.123**)	**<.001** *
Education level			
Illiteracy	Reference		
Primary school and below	**0.709**	**(0.542, 0.927**)	**.012**
Junior or senior high school	**0.484**	**(0.335, 0.699**)	**<.001** *
College and above	**0.233**	**(0.112, 0.483**)	**<.001** *
Marital status			
Married	Reference		
Widowed	0.792	(.0.560, 1.119)	.186
Others	1.581	(0.676, 3.697)	.290
Type of medical insurance			
Public medical	Reference		
Medical insurance	0.516	(0.072, 3.680)	.509
Business insurance	0.474	(0.057, 3.924)	.489
Others	0.448	(0.057, 3.539)	.448
Family income per month, CNY			
<1000	Reference		
1000-2000	**0.348**	**(0.107, 1.132**)	**.079**
2000 to 5000	0.389	(0.121, 1.245)	.112
>5000	**0.347**	**(0.108, 1.111**)	**.075**
Polypharmacy			
No	Reference		
Yes	**3.065**	**(2.343, 4.008**)	**<.001** *
Comorbidity			
No	**Reference**		
Yes	**2.326**	**(1.796, 3.012**)	**<.001** *
Pre-retirement occupation			
Institutions	Reference		
Foreign companies	0.955	(0.422, 2.160)	.912
Freelance business	0.878	(0.623, 1.238)	.459
Farmers.	0.837	(0.612, 1.146)	.268
Region			
County or town	Reference		
Rural	**1.770**	**(1.360, 2.303**)	**<.001** *
Smoking			
Always	Reference		
Occasional	0.790	(0.536, 1.165)	.235
Never	1.099	(0.753, 1.645)	.646
Drinking			
Always	Reference		
Occasional	1.005	(0.511, 1.977)	.998
Never	1.308	(0.645, 2.651)	.457
Traumatic event			
No	Reference		
Yes	0.712	(0.363, 2.396)	.323
History of falls			
No	Reference		
Yes	1.272	(0.725, 2.231)	.702
Surgical history.			
No	Reference		
Yes	1.305	(0.889, 1.918)	.174
Sarcopenia			
No	Reference		
Yes	**2.527**	**(1.976, 3.231**)	**<.001** *
Nutritional status			
Normal nutrition	Reference		
Risk of malnutrition	**1.906**	**(1.456, 2.497**)	**<.001** *
Malnutrition exists	**4.872**	**(3.109, 7.635**)	**<.001** *

Abbreviations: ADL = activities of daily living, BMI = body mass index, CI = confidence interval, GAD-7 = generalized anxiety scale, OR = odds ratio, PHQ-9 = depression screening scale, PSQI = Pittsburgh sleep quality index, SSRS = social support rating scale.

* *P* < .001.

### 3.4. Multivariable analysis

The multivariable analysis (Table 4) showed that age (OR = 1.13, 95% CI: 1.10–1.15, *P* < .001), GAD-7 (OR = 1.01, 95% CI: 1.03–1.11, *P* = .001), female sex (OR = 1.83, 95% CI: 1.10–1.16, *P* < .001), education level (college and above, OR = 0.27, OR = 0.12–0.64, *P* = .003), polypharmacy (OR = 2.29, 95% CI: 1.62–3.23, *P* < .001), comorbidity (OR = 1.93 95% CI: 1.37–2.71, *P < *.010), region (rural, OR = 1.77, 95% CI: 1.36–2.30, *P < *.001), sarcopenia (OR = 1.60, 95% CI: 1.16–2.19, *P* = .004), and nutritional status (risk of malnutrition, OR = 1.66, 95% CI: 1.17–2.35, *P* = .004; malnutrition exists, OR = 3.24, 95% CI: 1.85–5.83, *P* < .001) were independently associated with cognitive frailty. The results showed that literacy level and good nutritional status were protective factors for the development of cognitive frailty, while being female, multimorbidity, polypharmacy, sarcopenia, and remoteness were risk factors for the development of cognitive frailty.

**Table 4:** Multivariable logistic regression analysis.

Variable	OR	95% CI	*P*
Age	**1.131**	**(1.104, 1.158**)	**<.001** *
ADL	**0.966**	**(0.957, 0.975**)	**<.001** *
PSQI	1.028	(0.989, 1.069)	.165
GAD-7	**1.008**	**(1.033, 1.145**)	**.001**
PHQ-9	0.982	(0.953, 1.032)	.478
Sex (female vs male)	**1.830**	**(1.104, 1.158**)	**<.001** *
Education level			
Illiteracy	Reference		
Primary school and below	0.784	(0.549, 1.119)	.180
Junior or senior high school	0.658	(0.410, 1.058)	.084
College and above	**0.270**	**(0.115, 0.636**)	**.003**
Family income per month, CNY			
<1000	Reference		
1000 to 2000	0.288	(0.042, 1.990)	.207
2000 to 5000	0.187	(0.027, 1.281)	.088
>5000	0.158	(0.023, 1.078)	.060
Polypharmacy	**2.291**	**(1.624, 3.232**)	**<.001** *
Comorbidity	**1.928**	**(1.370, 2.713**)	**<.001** *
Region (rural vs county or town)	**1.667**	**(1.186, 2.343**)	**.003**
Sarcopenia	**1.596**	**(1.161, 2.193**)	**.004**
Nutritional status			
Normal nutrition	Reference		
Risk of malnutrition	**1.657**	**(1.169, 2.347**)	**.004**
Malnutrition exists	**3.24**	**(1.849, 5.831**)	**<.001** *

Abbreviations: ADL = activities of daily living, CI = confidence interval, GAD-7 = generalized anxiety scale, OR = odds ratio, PHQ-9 = depression screening scale, PSQI = Pittsburgh sleep quality index.

* *P* < .001.

### 3.5. Subgroup univariate regression analysis

Subgroup univariate regression analysis(Table 5) between different kinds of cardiovascular diseases and cognitive frailty showed that Coronary heart disease (OR = 1.638, 95% CI: 1.286, 2.087, *P* < .001), Arrhythmia (OR = 2.105, 95% CI: 1.296, 3.420, *P* = .003), Heart failure (OR = 2.891, 95% CI: 2.194, 3.809, *P* < .001), Hypertension (OR = 3.363, 95% CI: 1.501–7.563, *P* = .003).

**Table 5:** Subgroup univariate regression analysis between different cardiovascular diseases and cognitive frailty.

Variable	OR	95% CI	*P*
Coronary heart disease	**1.638**	**(1.286, 2.087**)	**<.001** *
Arrhythmia	**2.105**	**(1.296, 3.420**)	**.003**
Valvular heart disease	1.153	(0.803, 1.657)	.440
Heart failure	**2.891**	**(2.194, 3.809**)	**<.001** *
Hypertension	**3.363**	**(1.501, 7.563**)	**.003**

Abbreviations: CI = confidence interval, OR = odds ratio.

* *P* < .001.

## 4. Discussions

The prevalence of cognitive frailty is increasing in China^.[5,23]^ but there are disparities between hospitals and communities populations and across different diseases.^[24,25]^ Therefore, this study aimed to investigate the prevalence and risk factors of cardiovascular disease among elderly patients hospitalized in China. The results showed that the prevalence of cognitive frailty was 33.9%. Age, Anxiety, Female sex, Education level, Polypharmacy, Comorbidity, Region, Sarcopenia, and Nutritional status were independent risk factors for cognitive frailty.

Previous studies in China have shown that when the Fried phenotype was used to assess physical frailty and the Clinical Dementia Rating Scale was used to assess cognitive function, the prevalence of cognitive frailty was 26.7%.^[26]^ When using the Frailty Index to assess frailty status and the MMSE to assess cognitive function, the incidence of cognitive frailty was 25.4%.^[27]^ In this study, using the 2013 International Frailty Consensus Group diagnostic guidelines, the overall prevalence was 33.9%. The diverse definitions of cognitive frailty are a major issue in epidemiological studies of cognitive frailty.^[5]^In addition, The incidence of cognitive frailty in this study was higher than that of other diseases. This can be attributed to the fact that cardiovascular disease is both a risk factor and a contributing factor for cognitive frailty. Additionally, cardiovascular disease is also a risk factor for frailty. There is a strong association between cognitive impairment and frailty, with frailty being linked to higher rates of cardiovascular disease. Vascular risk factors play a significant role in the development of cognitive decline and dementia. Research^[28]^ indicates that coronary heart disease and cognitive function decline have a common basis. Risk factors for cardiovascular diseases, such as hypertension, hyperlipidemia, hyperglycemia, and heart failure, may initiate and promote the occurrence and progression of dementia.

This study reveals that patients with different cardiovascular diseases exhibit varying rates of cognitive frailty. Zhang Ning et al^[29]^ found that the prevalence of combined frailty in hospitalized elderly patients with coronary heart disease was 23.9% in a study of 364 patients with coronary heart disease hospitalized in the Department of Geriatrics and the Department of Cardiology at Peking Union Medical College Hospital. Coronary artery disease greatly increases the risk of cognitive impairment and dementia. The prevalence of cognitive impairment is high in patients with heart failure and may increase the ability to warn of early death in patients with heart failure. Jhasr et al^[26]^ in a study of patients with advanced heart failure, mainly in transplant centers, found that the prevalence of cognitive impairment was as high as 39.7%, and that the inclusion of cognitive dysfunction in the assessment of frailty could increase the ability to identify high-risk early deaths in cardiac transplant patients with advanced heart failure. The prevalence of cognitive frailty is high in patients with heart failure and may increase the ability to warn of early death in heart failure patients. One study^[30]^ found that frailty is 1 of the most important factors affecting the benefit of antihypertensive therapy in the elderly. The prevalence of frailty in elderly hypertensive patients is high, and it may increase the early warning of death in heart failure patients, and Fan Li et al^[31]^ conducted a study of 320 patients who were examined at the outpatient clinic of the General Hospital of the PLA. Fan Li et al^[31]^ found that the detection rate of frailty was 23.5 per cent in 320 elderly hypertensive patients attending the outpatient clinic of the General Hospital of the PLA: The detection rate of frailty was 23.1 per cent, of which 7.0 per cent was detected in elderly patients aged 65 to 80 years, and 7.0 per cent was detected in elderly patients with hypertension. The detection rate of frailty was 23.1 per cent, of which 7.0 per cent was found in 65 to 80-year-olds, and 32.0 per cent in ≥80 year olds. Reduced walking speed is an independent risk factor for falls in elderly hypertensive patients.^[32]^ The rate of detection was 7.0% in the elderly aged ≥ 80 years, and the rate of detection was 32.0%. The risk of cognitive impairment is increased by both high and low blood pressure in the elderly. The risk of cognitive impairment is increased by both high and low blood pressure in the elderly.^[32]^ Subgroup analyses showed that coronary heart disease, hypertension, arrhythmia and heart failure were closely associated with the occurrence of cognitive decline. Due to the amount of data and the cross-sectional design of the study, it cannot be directly concluded that heart valve disease is not an influential factor in the development of cognitive frailty.

This study emphasizes the significance of age as a risk factor for cognitive frailty in older cardiovascular patients who are hospitalized. This finding aligns with the research results of Chen et al.^[33]^ The correlation between age and cognitive decline can be attributed to degenerative changes in bodily functions, deterioration in social roles, and progressive aging of brain functions. Specifically, cognitive functions that are mediated by the frontal lobes tend to decline with age. Furthermore, this study also reveals that elderly female cardiovascular patients are more prone to experiencing cognitive frailty, which is consistent with the research findings of Chen et al.^[34]^ Additionally, the study highlights that polypharmacy and comorbidities contribute to the risk of cognitive frailty in cardiovascular patients, confirming the findings of Liu et al.^[35]^ One possible explanation is that cardiovascular patients often suffer from multiple comorbidities, such as hypertension, diabetes, and other chronic diseases. Elevated blood pressure and dyslipidemia can lead to thrombosis or reduced cardiac output, resulting in decreased cognitive abilities such as attention and executive function. Moreover, older adults who take multiple medications may experience symptoms such as memory loss and impaired intelligence, judgment, and language abilities.^[36]^

This study provides evidence that both sarcopenia and malnutrition are risk factors for cognitive frailty, consistent with previous research in community-dwelling older adults.^[25]^ Malnutrition exacerbates the loss of muscle strength and mass, leading to the development of sarcopenia. Sarcopenia, in turn, leads to frailty, which, coupled with nutritional deficiencies, leads to metabolic derangements that lead to cognitive impairment.^[37]^ Additionally, this study demonstrated a correlation between anxiety and cognitive function in older cardiovascular patients. The severity of cognitive decline is consistent with Wang Xiufang research results.^[38]^ Similarities in the physiological basis of cognitive frailty and anxiety, as well as the elevated expression of inflammatory factors in anxious and frail older adults, may explain this relationship.^[39]^In rural areas, compared with urban areas, education and education are relatively lower, and as the number of empty-nest elderly increases, rural elderly people have low social interaction and low awareness of health care, which may be the reason for the high incidence of cognitive frailty.

The present study has several limitations. Firstly, it only used a single definition of cognitive frailty, which restricts the ability to compare the prevalence with other regions of China and the world. Secondly, the study design was cross-sectional and lacked random sampling, which may affect the generalizability of the findings. Thirdly, the study population was limited to 1 tertiary hospital, further limiting the generalizability of the results. Additionally, the study variables lack objective laboratory and imaging indicators. Therefore, future research should prioritize conducting multi-center large-sample studies and longitudinal studies to gain a more comprehensive understanding of the risk factors for cognitive frailty in elderly cardiovascular patients.

## 5. Conclusion

This study highlights the high incidence of cognitive frailty in elderly cardiovascular patients who are hospitalized. The study identifies several independent influencing factors for cognitive frailty, including age, gender, education level, comorbidity, polypharmacy, insomnia, and anxiety. It is recommended that medical staff assess cognitive frailty early and implement targeted interventions based on the identified risk factors to prevent or reduce its occurrence.

## Acknowledgments

The authors thank for the support of participants and the help from the study staff, and volunteers.

## Author contributions

**Data curation:** Jinhua Guo, Yi Yang ,Yi Zhang.

**Formal analysis:** Jinhua Guo.

**Funding acquisition:** Jinhua Guo.

**Investigation:** Jinhua Guo, Yi Yang

**Methodology:** Jinhua Guo, Yi Zhang, lixia Lin.

**Resources:** lixia Lin.

**Supervision:** Yi Zhang.

**Writing – original draft:** Jinhua Guo.

**Writing – review & editing:** Tiemei Shen.
